# Causal‐Guided Ultra‐Long‐Term Time Series Forecasting Via Anticipated Covariates

**DOI:** 10.1002/advs.76201

**Published:** 2026-06-22

**Authors:** Jintong Zhao, Yufei Liu, Ruixi Huang, Zhongxue Gan, Siyang Leng

**Affiliations:** ^1^ Institute of AI and Robotics College of Intelligent Robotics and Advanced Manufacturing Fudan University Shanghai China; ^2^ Research Institute of Intelligent Complex Systems Fudan University Shanghai China

**Keywords:** causality, long short‐term memory, long‐term prediction, nonlinear dynamical systems, time series, transformer

## Abstract

Time series forecasting aims to discern latent patterns and predict future states. The incorporation of covariates, such as seasonal information, has effectively improved medium‐ to long‐term forecasting accuracy by capturing cyclical trends. However, the gradual accumulation of errors continues to constrain performance over ultra‐long horizons. Often treated as unknown, information from the future remains underutilized. In this work, we demonstrate that in a coupled dynamical system, providing the future state of the effect X enables accurate forecasting of the cause Y for thousands of timesteps. The forecasting error remains bounded even in the presence of an unobserved variable Z driving the target (i.e., Z→Y→X), confirming that future effect information inherently stabilizes and guides the trajectory of present causes. In light of this, we propose a time series forecasting paradigm that introduces *anticipated covariates* to represent such known future states. We validate this finding across several widely adopted benchmarks. Under suitable conditions, ultra‐long‐term predictions become feasible with forecast errors substantially reduced. We expect the anticipated covariates paradigm to be a powerful tool for situations constrained by high data costs, limited historical data, or unobservable causal factors, and to prompt a re‐evaluation of reverse time‐dependent causality.

## Graphical abstract

Often treated as unknown, information from the future remains underutilized.We demonstrate that in a coupled dynamical system, providing the future state of the effect enables accurate forecasting of the cause for a long timesteps. A time series forecasting paradigm that introduces anticipated covariates to represent such known future states is proposed to achieve accurate ultra ‐ long ‐ term prediction.

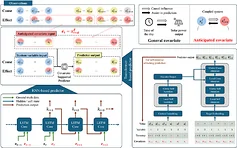

## 1 Introduction

Humanity has never ceased its endeavors to predict the future. This endeavor has crystallized into the discipline of time series forecasting, which involves analyzing temporal patterns in sensor readings or quantified metrics to extrapolate future states or recover missing temporal segments. Through extensive interdisciplinary research into the mechanisms governing nature and human society, researchers have developed comprehensive theoretical framework and empirical insights. These intellectual achievements have enabled transformative applications such as accurate precipitation probability estimations for daily planning and precise orbital control of artificial satellites. Predictions of natural hazards and epidemic diseases have reduced losses in human lives and property [1, 2, 3]. Forecasts of industrial processes and supply chains have improved the operational efficiency of societal systems [4, 5]. Projections of solar energy availability and atmospheric ${\mathrm{CO}}_{2}$ concentrations have progressively enhanced living conditions [6, 7]. Recent advancements in data‐driven machine learning methodologies have demonstrated remarkable predictive capabilities. Neural network‐based predictors have surpassed conventional numerical computation methods in performance metrics. For instance, deep learning architectures have outperformed physics‐based simulations in meteorological forecasting [8, 9, 10], while convolutional neural networks trained on Gramian angular fields have demonstrated efficacy in predicting U.S. market trends [11]. Significant progress has also been made in environmental monitoring and natural disaster prediction [12, 13, 14].

For general sequence prediction tasks, recurrent neural networks (RNNs) have long been capable of handling broader time series structures due to their characteristic of circulating states within the network. Specifically, Long Short‐Term Memory (LSTM) networks and their variants have demonstrated significant efficacy in various domains. For instance, an enhanced LSTM optimized via the Improved Sparrow Search Algorithm has significantly improved precision in building air conditioning load forecasting, demonstrating promising application prospects in advancing energy efficiency management [15]. Similarly, in the domain of short‐term wind power forecasting, novel LSTM architectures incorporating multi‐task temporal feature attention mechanisms have been proven effective using data from the National Renewable Energy Laboratory [16]. Further validation is found in hybrid wind speed prediction models, which outperformed baselines in experiments on seasonal wind speed datasets from the Dong Xinzhuang wind farm in Shaanxi, China [17]. As forecasting tasks grow in complexity–often involving intricate spatiotemporal interactions across heterogeneous variables–modern architectures must concurrently capture temporal dynamics and cross‐variable dependencies. Attention‐based Transformer models excel in this regime. For urban mobility optimization, the Spatiotemporal Fusion Transformer tackles large‐scale traffic flow prediction with exceptional efficiency, seasonal pattern recognition, and robustness [18]. In tourism analytics, Transformer‐based demand forecasting deciphers latent factors influencing travel patterns, offering actionable insights for resource allocation [19]. Moreover, novel Transformer‐LSTM hybrids have advanced intelligent pump station operations by strengthening inter‐factor correlations, thereby enhancing flood mitigation strategies under extreme weather events [20]. Real‐world forecasting targets exhibit multidimensional dependencies influenced by various external factors. Effective predictive modeling necessitates systematic integration of known influential covariates – auxiliary information not subject to prediction, which may manifest as static data (e.g., entity‐specific attributes) or dynamic time series (e.g., diurnal cycles, seasonal patterns). In recent years, numerous forecasting models have extended their consideration of auxiliary features [21, 22, 23, 24].

However, despite substantial research focused on exploring the underlying causes of forecasting targets and developing approaches for extracting and utilizing such information, achieving ultra‐long‐term forecasting remains a significant challenge due to error accumulation. There is still a lack of paradigms that start from known outcomes influenced by the forecasting targets, use these results to infer the causes necessary to generate them, and leverage future information to guide predictions. Causality inference and time series forecasting are naturally connected [25]. Both widely used Granger causality [26] and transfer entropy [27] infer causal direction by measuring “improvement in predictive power”, translating causality into the difference in predictive value of historical information between variables. Granger causality relies on linear models, testing statistical significance by comparing prediction errors of regression models with or without X to determine causation. Transfer entropy, grounded in information theory, quantifies nonlinear causal information flow by calculating the reduction in conditional entropy of Y’s future uncertainty when incorporating X’s historical data. Both emphasize that “if X’s past systematically enhances predictions of Y, a statistical causal link exists”. Extending such conclusions, when the effect variable Y undergoes dynamic trajectory changes due to the influence of the cause variable X, these changes inherently encode the state information of X at past moments.

In this paper, we propose an anticipated covariates empowered time series forecasting paradigm, which incorporates elements with known future states from coupled systems as covariates input to common neural network forecasting algorithms. We term these “anticipated covariates” to reflect their role in capturing the “anticipation of the cause” embedded in the effect. The proposed paradigm enables seamless enhancement of conventional predictive neural networks. Taking LSTM and Spacetimeformer as representative cases, we validate the paradigm's exceptional performance in ultra‐long‐term forecasting across multiple real‐world datasets. Through unrestricted‐length forward prediction without accuracy degradation, one can conveniently reconstruct the complete dataset panorama using partial time series segments from the dataset. Extended experiments demonstrate the practical value of incorporating anticipated covariates in challenging scenarios where critical elements affecting forecasting targets remain unobservable. The utilization of anticipated covariates not only bridges accurate ultra‐long‐term forecasting to practical implementation, but also provides novel insights into how machine learning systems might appropriately leverage future information. By rethinking the temporal dependence of cause and effect, this approach further advances our in‐depth understanding of causality and the cross‐fertilization of causality and machine learning.

## 2 Results

### 2.1 Anticipated Covariates

The prediction of multivariate time series $\left\{x_{t}\right\}$ can be formalized as a sequence‐to‐sequence task, where a predictor receives an input sequence $\left\{x_{t-s},x_{t-s+1},\text{…},x_{t}\right\}$ and generates an output sequence $\left\{{\hat{x}}_{t+1},{\hat{x}}_{t+2},\text{…},{\hat{x}}_{t+h}\right\}$ that approximates the ground truth $\left\{x_{t+1},x_{t+2},\text{…},x_{t+h}\right\}$. Variables that participate in both input and output sequences are termed *forecasting variables* for clarity, and their corresponding time series are designated as forecasting sequences. Generally, forecasting variables align with the variables involved in the underlying coupled system. The model aims to capture spatiotemporal dependencies within these sequences to forecast the system's future states. Real‐world systems are rarely autonomous. Thus, incorporating relevant external information is critical for enhancing prediction accuracy. For example, recent e‐commerce activity can inform sales forecasts, observed rainfall and weather forecasts can improve predictions for hydropower and solar energy generation, and awareness of upcoming holidays can refine demand forecasting. In practice, when certain future states are regarded as affecting the targets, encoding these known states as covariates provides a rational and effective strategy. In this context, *covariates* refer to variables that contribute to the forecasting model by being additional inputs but not part of the outputs, with their states $\left\{e_{t-s},e_{t-s+1},\text{…},e_{t+h}\right\}$ aligned to corresponding input and output timesteps. Notably, the commonly used covariates are selected on their effect on the forecasting variables and usually represent a different physical or virtual meaning from that of the forecasting variables. Here, we introduce *anticipated covariates*, which represent a special case where, in the coupled system, the future states of the effect variable affected by the prediction target serve as the covariate. The temporal offset $d=t_{2}-t_{1}$, where $t_{2}$ denotes the corresponding timestep of the anticipated covariate and $t_{1}$ the prediction timestep, is defined as *future info delay*. That is, for anticipated covariates, $\left\{e_{t-s}^{i},e_{t-s+1}^{i},\text{…},e_{t+h}^{i}\right\}$ is identical to $\left\{x_{t-s+d}^{j},x_{t-s+d+1}^{j},\text{…},x_{t+h+d}^{j}\right\}$, where $\left\{e_{t}^{i}\right\}$ denotes the sequence of i‐th anticipated covariate input to the model and $\left\{x_{t}^{j}\right\}$ represents the sequence of the corresponding forecasting variable.

To ensure complete accessibility of the system by the predictor and the flexible adaptability of the anticipated covariates paradigm, it is not required that variables serving as anticipated covariates be excluded from the forecasting variables. Introducing future states of forecasting variables as covariates is analogous to providing the model with “answers” to future queries. In practice, however, only a specific subset of the system variables is of primary interest and is naturally different from those forecasting variables serving as anticipated covariates. We use *target variables* to denote this subset of forecasting variables against which forecasting performance is evaluated for fairness considerations. Importantly, this paradigm diverges from iterative short‐term forecasting with ground truth resetting. Synthetic experiments confirm that performance improvements arise not from leaking future information but from leveraging causal dependencies: using effects (downstream variables influenced by target variables) as anticipated covariates enables infinite‐step accurate prediction, whereas using causes (upstream drivers) fails to enhance accuracy. Given the widespread adaptation of contemporary forecasting models to input covariates, particularly their inherent support for heterogeneous input‐output dimensionality, integrating these architectures into the anticipated covariate paradigm is operationally straightforward. To demonstrate the paradigm's versatility, we implement it in two representative architectures: the Long Short‐Term Memory network, an RNN backbone, and the Spacetimeformer model, which leverages attention mechanisms, both of which are extensively adopted in time series forecasting research.

Each dataset used is partitioned into 70% training, 10% validation, and 20% test sets. Model performance is evaluated using two strategies: Standard Testing (ST): Sliding windows split the test set into s‐step input and h‐step output segments; predictions are compared to ground truth via MAE and MSE. Ultra‐Long‐Term Forecasting (ULTF): The entire test set is processed in a single window, where the model autoregressively iterates using its prior outputs as inputs. Errors are reported in both unnormalized (raw scale) and normalized (using robust scaler to removes the median and scale the data according to the quantile range) forms for cross‐comparison. To avoid the influence of the accurate future state input to the model on the metrics, we calculate forecast errors only considering target variables that do not contain anticipated covariates in the meantime.

### 2.2 Real‐World Datasets

#### 2.2.1 Weather

The dataset comprises Local Climatological Data provided by the National Centers for Environmental Information, featuring high‐temporal‐resolution climate parameters recorded at thousands of global weather stations [28]. As a popular benchmark for medium‐ and long‐term time series forecasting tasks, it contains hourly observations of three temperature metrics: dry‐bulb temperature (ambient air temperature), wet‐bulb temperature (evaporation‐cooled temperature), and dew‐point temperature (air saturation temperature), each recorded in both Fahrenheit and Celsius. Beyond temperature data, additional variables include relative humidity, wind speed, station‐observed atmospheric pressure, and altimeter readings. The study investigates whether non‐thermal sequences can serve as anticipated covariates to accurately predict all three temperature types, thereby enabling holistic dataset reconstruction from partial observations. To eliminate interference from high similarity between Fahrenheit and Celsius records, we use Celsius‐scale data for ultra‐long‐term forecasting only. Experiment results in Figure 2a demonstrate that Spacetimeformer achieves approximately 7000 timesteps self‐iterative predictions using only a 60 timesteps context window combined with future information from four non‐thermal covariates. Throughout the forecasting process, prediction errors remain within acceptable bounds without increasing divergence from ground truth over extended horizons.

**FIGURE 1:**
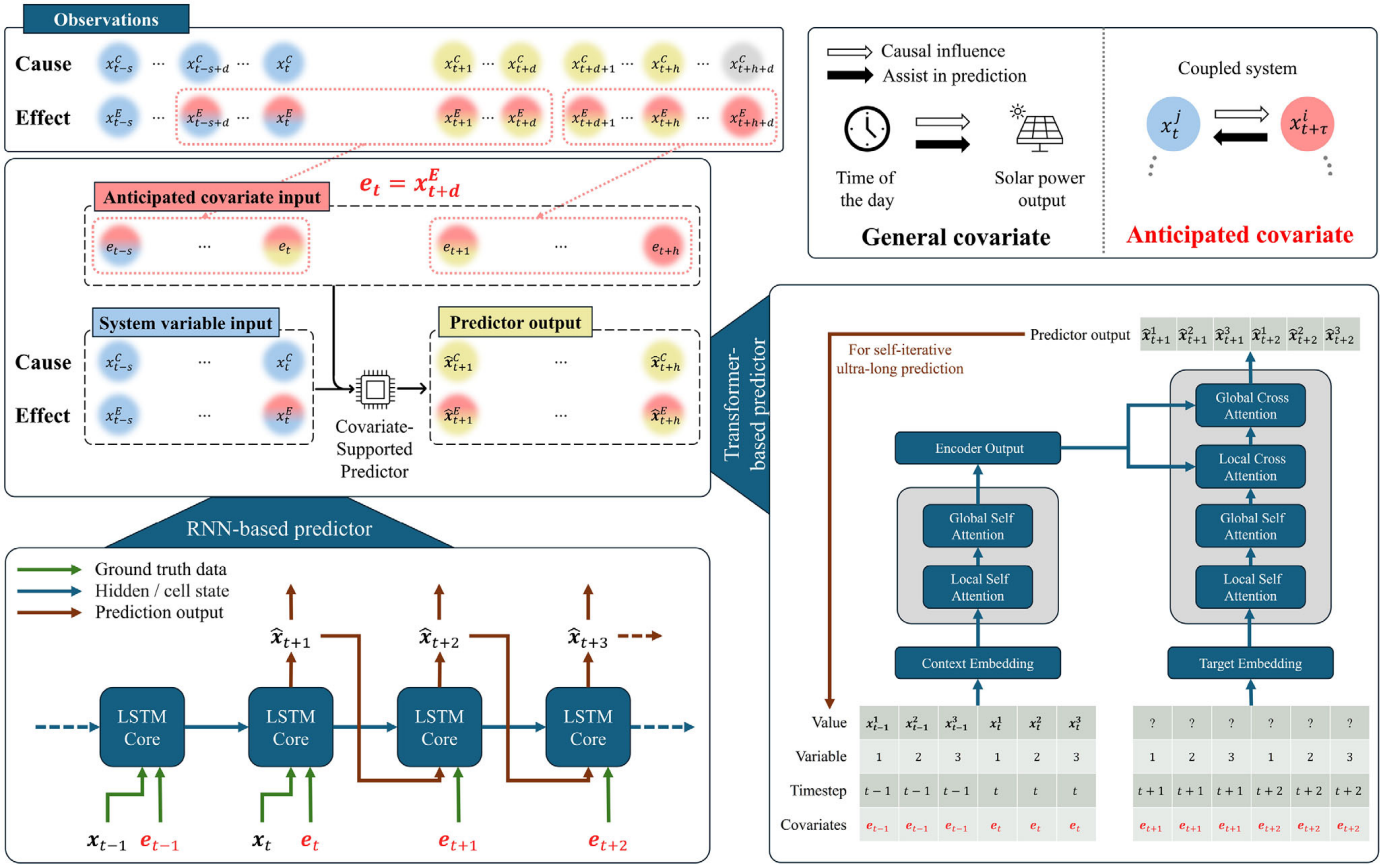
Framework of the anticipated covariate paradigm. Anticipated covariates are derived from the future states of the effects within a coupled system, sharing the same format as general covariates. The schematic illustrates the data processing workflow and how these covariates are integrated into representative architectures to guide ultra‐long‐term forecasting of the target variables ‐ the causes.

**FIGURE 2:**
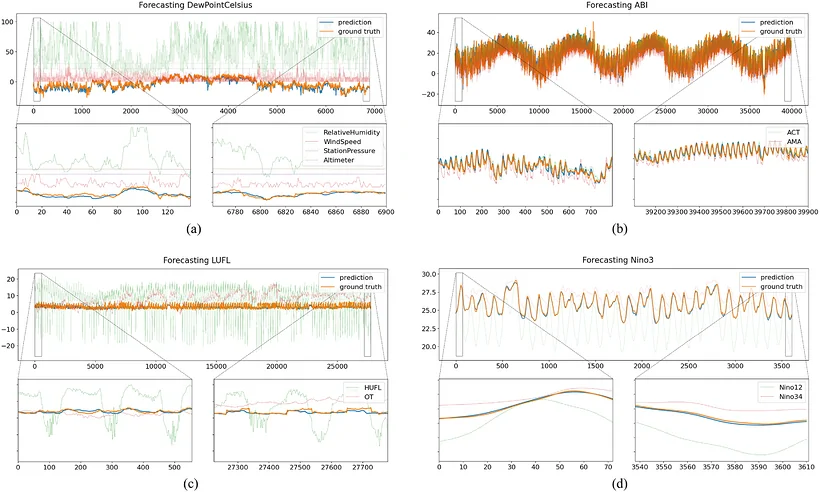
Ultra‐long‐term forecasting performance on real‐world datasets. (a) Weather, (b) ASOS, (c) ETTm1, and (d) ENSO. Top panels show global trajectories, while bottom panels highlight zoomed‐in details at the beginning and end of the forecasting horizon. Solid lines represent predictions vs. ground truth; dashed lines indicate the input anticipated covariates. All models are evaluated with a context length s∈[40,60], while the prediction length h encompasses the entire remaining duration of the test set.

#### 2.2.2 NY‐TX Weather

This dataset incorporates comprehensive meteorological benchmark data from six geographical locations collected through the National Weather Service Automated Surface Observing System (ASOS) [24]. Spanning 72 years (1949–2021) of hourly temperature observations with exceptionally long temporal coverage, it provides an ideal testbed for evaluating long‐sequence spatiotemporal forecasting models. Three locations are situated in central Texas, while the remaining three are hundreds of miles away in eastern New York. Adjacent geographical positioning naturally induces pairwise circular causal topologies among time series, whereas sequences of distant positionings exhibit negligible mutual influence. The inherent dynamical interactivity combined with region‐specific meteorological influences makes this dataset particularly suited for validating the critical role of future‐informed anticipated covariates. Using airport codes as identifiers, we select three central Texas locations for validation: ABI (target variable) paired with ACT and AMA (anticipated covariates). As shown in Figure 2b, the Spacetimeformer guided by anticipated covariates not only enables ultra‐long‐term predictions but also demonstrates reduced test errors compared to baseline approaches (overall unnormalized MSE 7.97 vs. 12.49 for data from Ref. [24]), confirming enhanced accuracy in medium‐to‐long‐term forecasting.

#### 2.2.3 ETTm1

The electricity distribution problem refers to how power grids manage electricity allocation to different user areas based on sequentially changing demand. However, predicting future demand for specific user areas is challenging due to varying factors such as workdays, holidays, seasons, weather, temperature, etc. The dataset records six different kind of external loads, along with the oil temperature of transformers, which effectively reflects their operational status [28]. By combining specific load and oil temperature data, we achieved accurate ultra‐long‐term forecasting for another load with a clearly differentiated trajectory, as shown in Figure 2c. Notably, to focus on the role of anticipated covariates, we omitted date‐related information compared to the original Spacetimeformer model.

#### 2.2.4 El Niño‐Southern Oscillation

The El Niño phenomenon manifests as a natural climatic event characterized by anomalous sea surface temperature (SST) elevations in the equatorial central‐eastern Pacific Ocean, predominantly spanning key monitoring regions (Niño zones: Niño 1+2, Niño 3, Niño 3.4, and Niño 4) [29]. This oceanic warming is typically accompanied by atmospheric circulation anomalies, triggering global extreme weather patterns. The El Niño‐Southern Oscillation (ENSO) system constitutes a coupled ocean‐atmosphere interaction, integrating the oceanic phases of El Niño/La Niña (cool phase) with the atmospheric Southern Oscillation (east‐west pressure oscillations). These components collectively drive ENSO cycles through dynamic air‐sea feedback mechanisms. The demarcation of Niño regions, particularly the Niño 3.4 zone, serves as a critical metric for ENSO monitoring, where the magnitude and spatial extent of SST anomalies directly determine the classification and intensity of ENSO events. For instance, sustained warming in the Niño 3.4 region is a defining criterion for El Niño identification, while distinct anomaly patterns across eastern (e.g., Niño 3) and central Pacific regions (e.g., Niño 4) differentiate ENSO spatial modalities (eastern vs. central Pacific types), thereby modulating their global climatic impacts. Predictive modeling of SST anomalies in these Niño regions enables multi‐month anticipation of ENSO evolution, offering vital scientific insights for agricultural planning, water resource management, and disaster mitigation. Such forecasts empower policymakers to develop adaptive strategies, reducing socioeconomic vulnerabilities to ENSO‐driven hazards like floods and droughts. Studies [29, 30] have confirmed the coupling relationships among the adjoining Niño regions' temporal dynamics. Leveraging this interdependence, our paradigm employs adjacent regions as anticipated covariates to achieve ultra‐long‐term forecasting, as demonstrated in Figure 2d.

See Table 1 for detailed real‐world dataset results with more combinations.

**TABLE 1:** Summary of real‐world datasets results with different settings.

Dataset	Anticipated covariates	Target variables	Method	Metrics (normalized / unnormalized)			
				ST		ULTF	
				MAE	MSE	MAE	MSE
Weather	RH, WS, SP, Al	DBC, DPC, WBC	STF	0.163 / 1.909	0.051 / 6.880	0.305 / 3.547	0.162 / 3.547
Weather	RH, WS, SP, Al	DBF, WBF, DPF	STF	0.169 / 3.560	0.063 / 27.230	0.309 / 6.514	0.174 / 6.514
Weather	RH, WS, SP, Al	DBC, DPC, WBC	LSTM	0.129 / 1.518	0.036 / 4.942	0.245 / 2.893	0.103 / 2.893
ASOS	ACT, AMA	ABI	STF	0.116 / 1.681	0.024 / 4.996	0.122 / 1.777	0.026 / 1.777
ASOS	AMA	ABI, ACT	STF	0.152 / 2.158	0.041 / 8.196	0.171 / 2.430	0.049 / 2.430
ASOS	ACT	ABI, AMA	STF	0.143 / 2.243	0.037 / 9.341	0.164 / 2.581	0.046 / 2.581
ASOS	ABI	ACT, AMA	STF	0.131 / 2.011	0.031 / 7.401	0.143 / 2.205	0.036 / 2.205
ASOS	ACT, AMA	ABI	LSTM	0.117 / 1.699	0.024 / 5.052	0.118 / 1.710	0.024 / 1.710
ETTm1	HUFL, OT	LUFL	STF	0.412 / 0.557	0.355 / 0.650	0.509 / 0.688	0.501 / 0.688
ETTm1	HUFL, OT	LUFL	LSTM	0.446 / 0.603	0.374 / 0.685	0.438 / 0.593	0.363 / 0.593
ENSO	Nino3	Nino12	STF	0.132 / 0.515	0.030 / 0.453	0.133 / 0.519	0.030 / 0.519
ENSO	Nino3	Nino12	LSTM	0.127 / 0.494	0.028 / 0.418	0.125 / 0.487	0.027 / 0.487
ENSO	Nino12, Nino34	Nino3	LSTM	0.067 / 0.127	0.007 / 0.025	0.065 / 0.123	0.006 / 0.123
ENSO	Nino3, Nino4	Nino34	LSTM	0.112 / 0.140	0.018 / 0.029	0.112 / 0.140	0.018 / 0.140
ENSO	Nino34	Nino4	LSTM	0.303 / 0.259	0.124 / 0.091	0.313 / 0.267	0.131 / 0.267

*Note*: All metrics are calculated from target variables. ST: Standard Testing, ULTF: Ultra‐Long‐Term Forecasting, STF: Spacetimeformer, RH: Relative Humidity, WS: Wind Speed, SP: Station Pressure, Al: Altimeter, DBC: Dry Bulb Celsius, DPC: Dew Point Celsius, WBC: Wet Bulb Celsius, DBF: Dry Bulb Fahrenheit, WBF: Wet Bulb Fahrenheit, DPF: Dew Point Fahrenheit, HUFL: High UseFul Load, OT: Oil Temperature, LUFL: Low UseFul Load. Datasets abbreviations follow original definitions.

### 2.3 Synthetic Datasets

To further investigate the reverse temporal dependence arising from the significant impact of future information on predictions, while considering that uncorrelated additional sequences should not exert any influence on the forecasting of target sequences, we conduct our analysis through the lens of causal relationships in coupled systems. We consider a network comprising N nodes, each constituting a Lorenz system with parameter heterogeneity. The internal dynamics of the k‐th node are governed by:

(1) $$\begin{array}{cc} \frac{dx^{k}}{dt} & =-10\left[x^{k}-y^{k}+c\sum_{l=1}^{N}a_{kl}^{\left(x,y\right)}\left(y^{l}-y^{k}\right)\right]+\varepsilon_{t}^{k,x},\\ \frac{dy^{k}}{dt} & =28\left(1+\rho_{h}^{k}\right)x^{k}-y^{k}-x^{k}z^{k}+\varepsilon_{t}^{k,y},\\ \frac{dz^{k}}{dt} & =-\left(8/3\right)z^{k}+x^{k}y^{k}+\varepsilon_{t}^{k,z}, \end{array}$$

where c=0.3 regulates the coupling strength, and $a_{kl}^{\left(x,y\right)}$ is a binary variable with possible values 0 and 1, controlling the directed interaction from dimension y of Node l to dimension x of Node k. The adjacency matrix $A=\left[a_{ij}^{\left(x,y\right)}\right]\left(i,j=1,2,\text{…},N\right)$ encodes the causal topology. Heterogeneity is introduced via $\rho_{h}^{k}$, randomly sampled from $\left[-\rho_{h},\rho_{h}\right]$ with $\rho_{h}=0.06$. Dynamical noise is modeled by additive independent white noise terms $\varepsilon_{t}^{k,d},d=\left(x,y,z\right)$, characterized by zero mean and standard deviation $\sigma_{\mathrm{dyn}}=10^{-4}$.

A fundamental tenet in causal research posits that incorporating historical causal information into predictive models enhances outcome forecasting. Correspondingly, the integration of future outcome information should theoretically enable retrospective inference regarding the causal states that produce such outcomes. To validate which part of a coupled system qualify as a helpful covariate ‐ whether the cause, effect, or just anyone ‐ a minimalistic causal topology comprising two nodes with a unidirectional causal link from Node 2 to Node 1 is employed.

**FIGURE 3:**
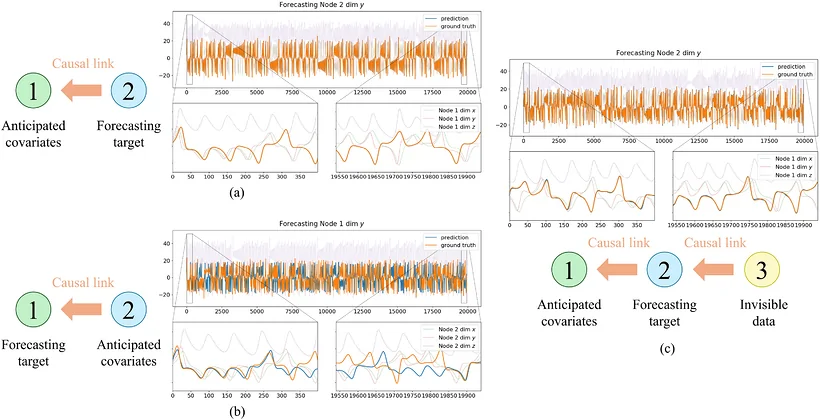
LSTM‐based ultra‐long‐term forecasting on coupled Lorenz systems. (a) Two‐node topology with a 2→1 causal link; using future effect information as an anticipated covariate enables high‐precision forecasting of the cause for 20 000 steps ($\mathrm{MSE}=7.83\times 10^{-6}$). (b) Inverse case where the cause serves as the covariate; accuracy degrades after ∼300 steps (MSE=0.43), reflecting causal asymmetry. (c) Robustness under unobserved drivers (Node 3); the model accurately recovers the target (Node 2) by leveraging its downstream effect (Node 1) as the anticipated covariate (MSE=0.0045).

As demonstrated by the results in Figure 3, where Node 1 and Node 2 are alternately employed as the covariate and target variable, accurate ultra‐long‐term forecasting is achieved only when utilizing the effect as the anticipated covariate. Under noise‐free observation conditions, the model achieved exceptional prediction accuracy over nearly 20 000 timesteps, with test set length being the constraint on prediction duration. Notably, while ultra‐long‐term forecasting maintained characteristic trajectories of the Lorenz system ‐ indicating adequate model training ‐ the utilization of future cause states as anticipated covariates shown in Figure 3b precipitated significant deviation from ground truth after approximately 300 timesteps, mirroring the performance baseline without covariates. This discrepancy reaffirms the temporal asymmetry inherent in causal relationships. Effects yet to come hold as much value as causes from the past.

Surprisingly, when the target variables are influenced by unobservable external factors while simultaneously driving third‐party effects, future information of such effects can effectively substitute for historical causes. Conventionally, forecasting a target system requires collecting all potential drivers and modeling their dynamical interactions. However, our results suggest that if the relationship between the target and its subsequent effects is accurately modeled, the explicit inclusion of the target's original causes becomes unnecessary. In this context, the target's state is implicitly constrained by its effects: specifically, only certain antecedent states can generate the observed future outcomes. Experiment results in Figure 3c confirm this, demonstrating that future effect information provides a sufficient substrate for accurate ultra‐long‐term forecasting even when critical drivers (Node 3) are hidden. In our configuration, Node 3 ‐ causally influencing the forecasting target Node 2 ‐ is exclusively employed for time series generation, remaining inaccessible during both training and prediction phases. Within this constrained forecasting scenario, Node 1 as the anticipated covariate provided sufficient informational substrate. The LSTM‐based predictor successfully deciphers the dynamical relationship dictating what antecedent states must arise to lead to such future consequences, enabling accurate ultra‐long‐term forecasting.

In fact, the forecasting variable corresponding to the anticipated covariate, Node 1, is not necessary to be inputted as a forecasting variable either. To fully eliminate the advantage that future information confers on the effect node during the forecasting process, an additional experiment is conducted under the setup shown in Figure 3c, while Node 1 is excluded from both the input and output of the predictor, i.e., only serves as anticipated covariate, and the forecast variable includes only Node 2. To be more specific, let $X_{1}$ denote the effect, Node 1, and $X_{2}$ the target cause, Node 2. The pre‐hiding setup as shown in Figure 3c can be described as

$$\left({\hat{X}}_{2,\;t+1:t+h},\;{\hat{X}}_{1,\;t+1:t+h}\right)\;=\;N\left(\underset{\text{FV history}}{\underset{︸}{X_{2,\;t-s:t},\;X_{1,\;t-s:t}}},\;\underset{\text{AC future}}{\underset{︸}{X_{1,\;t-s+d:t+h+d}}}\right),$$

where N is the neural network predictor, $X_{k,\;t_{1}:t_{2}}$ indicates the corresponding sequence of node $X_{k}$ $\left\{X_{k,t_{1}},X_{k,t_{1}+1},\text{…},X_{k,t_{2}}\right\}$, FV: forecasting variables and AC: anticipated covariates. While the post‐hiding setup where $X_{1}$ is removed from the forecasting variables but its future sequence is still retained as an anticipated covariate can be described as

$${\hat{X}}_{2,\;t+1:t+h}\;=\;N\left(\underset{\text{FV history}}{\underset{︸}{X_{2,\;t-s:t}}},\;\underset{\text{AC future}}{\underset{︸}{X_{1,\;t-s+d:t+h+d}}}\right).$$

The ultra‐long‐term forecasting accuracy of Node 2 after hiding Node 1 is almost indistinguishable from that before hiding (unnormalized ULTF MSE: 0.486 vs. 0.497). This is further evidence that anticipated covariates do NOT work by improving the accuracy of their corresponding forecasting variables and thus the global accuracy. This counterintuitive phenomenon provides new insights into the traditional framework of causal reasoning, demonstrating the superior performance of effect‐oriented modeling approaches.

Together, these results provide three levels of evidence distinguishing our paradigm from future information leakage. First, the trajectories of the anticipated covariate and the target variables are visibly distinct in shape, scale, and physical meaning, ruling out the possibility that the covariate is merely a delayed or similar copy of the target. Second, a clear causal asymmetry is observed: using the effect to predict the cause enables ultra‐long accurate forecasting, whereas using the cause to predict the effect fails rapidly. If this were simple future leakage, the reverse direction would also work, yet it does not. Third, even when the effect variable is completely excluded from the predictor's inputs and outputs, i.e., the model never sees or predicts the effect and only receives its future values as an external covariate, the ultra‐long forecasting of the cause remains accurate. This confirms that the anticipated covariate provides genuinely new information to its upstream cause, leveraging causal dependency rather than temporal shortcuts.

### 2.4 Further Experiments

#### 2.4.1 Observation Noise

**FIGURE 4:**
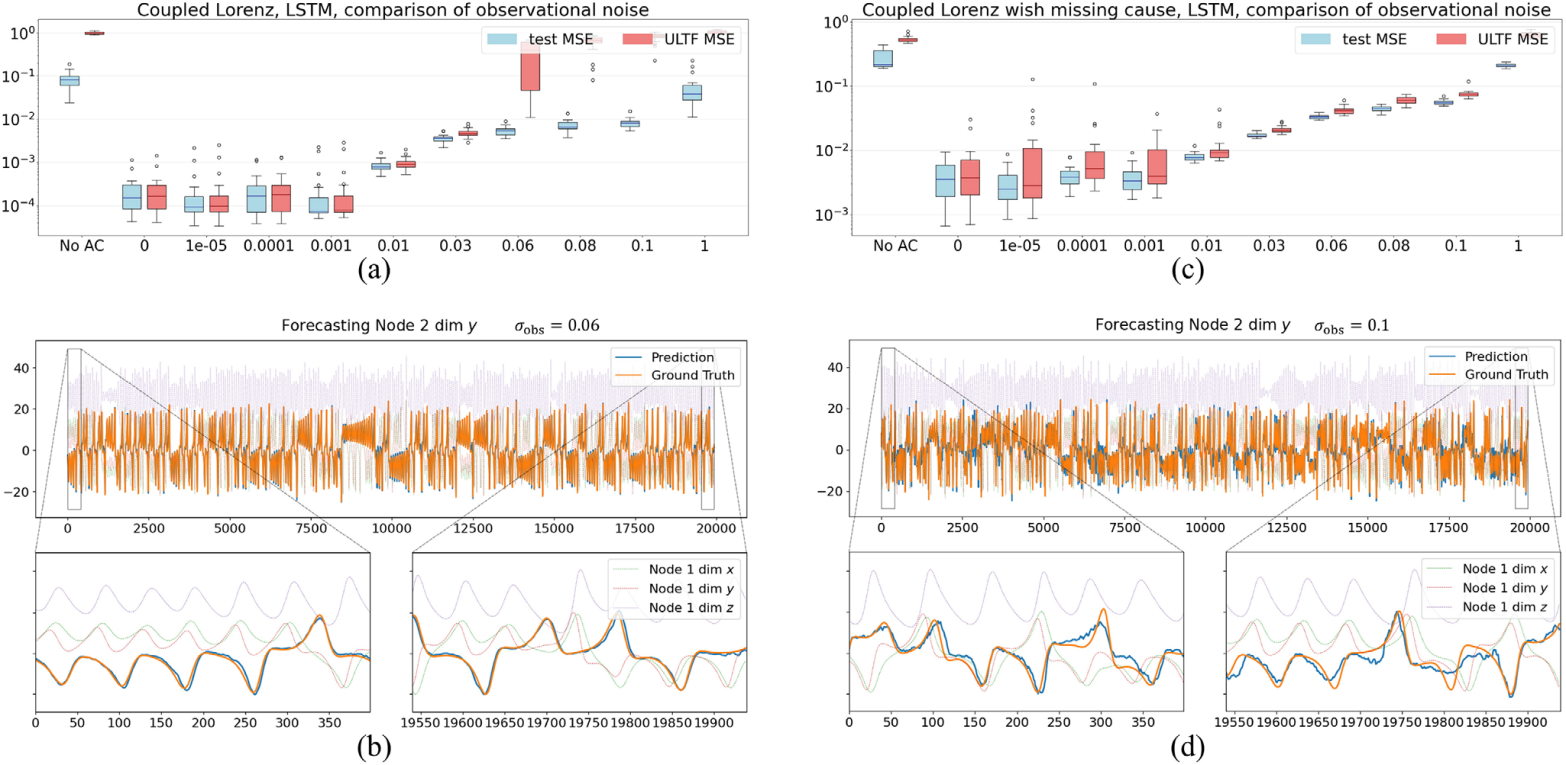
Sensitivity analysis of observational noise. Compare the errors of performing multiple long‐term predictions on the test set and a single ultra‐long‐term forecasting using the entire test set. Results without any anticipated covariates serve as baselines. The context length s is set to 60. Each option is repeated 20 times.

The utilization of anticipated covariates necessitates targeted acquisition of future states relative to prediction timestamps, which may originate from existing predictors, costly or difficult‐to‐acquire sensor data, or precision‐limited historical records. To further validate the practical utility of our proposed paradigm, we simulate these operational constraints by introducing observational noise to anticipated covariates. Gaussian noise with zero mean and varying standard deviations ($\sigma_{\mathrm{obs}}$) is applied to normalized time series during numerical experiments. Using synthetic data generated through the previously described causal topology ‐ “Coupled Lorenz” from Figure 3a and “Coupled Lorenz with missing cause” from Figure 3c, we systematically evaluate the robustness against unreliable future information and its performance under missing critical components. As shown in Figure 4, the system maintains ultra‐long‐term forecasting accuracy even at $\sigma_{\mathrm{obs}}=0.06$, where prediction errors remain below test errors of baseline and trajectories show no significant deviation from ground truth. On the other hand, the presence of hidden influential factors brings substantial training challenges. The model fails to capture underlying variation patterns in target sequences, resulting in inaccurate predictions even within 80‐step training windows (much larger test error of no anticipated covariates setting compared with standard “Coupled Lorenz”). While observational noise introduces minor oscillations in forecasting errors, the predicted trajectories remain centered around the ground truth, ensuring robust ultra‐long‐term trend estimation.

#### 2.4.2 Future Info Delay

**FIGURE 5:**
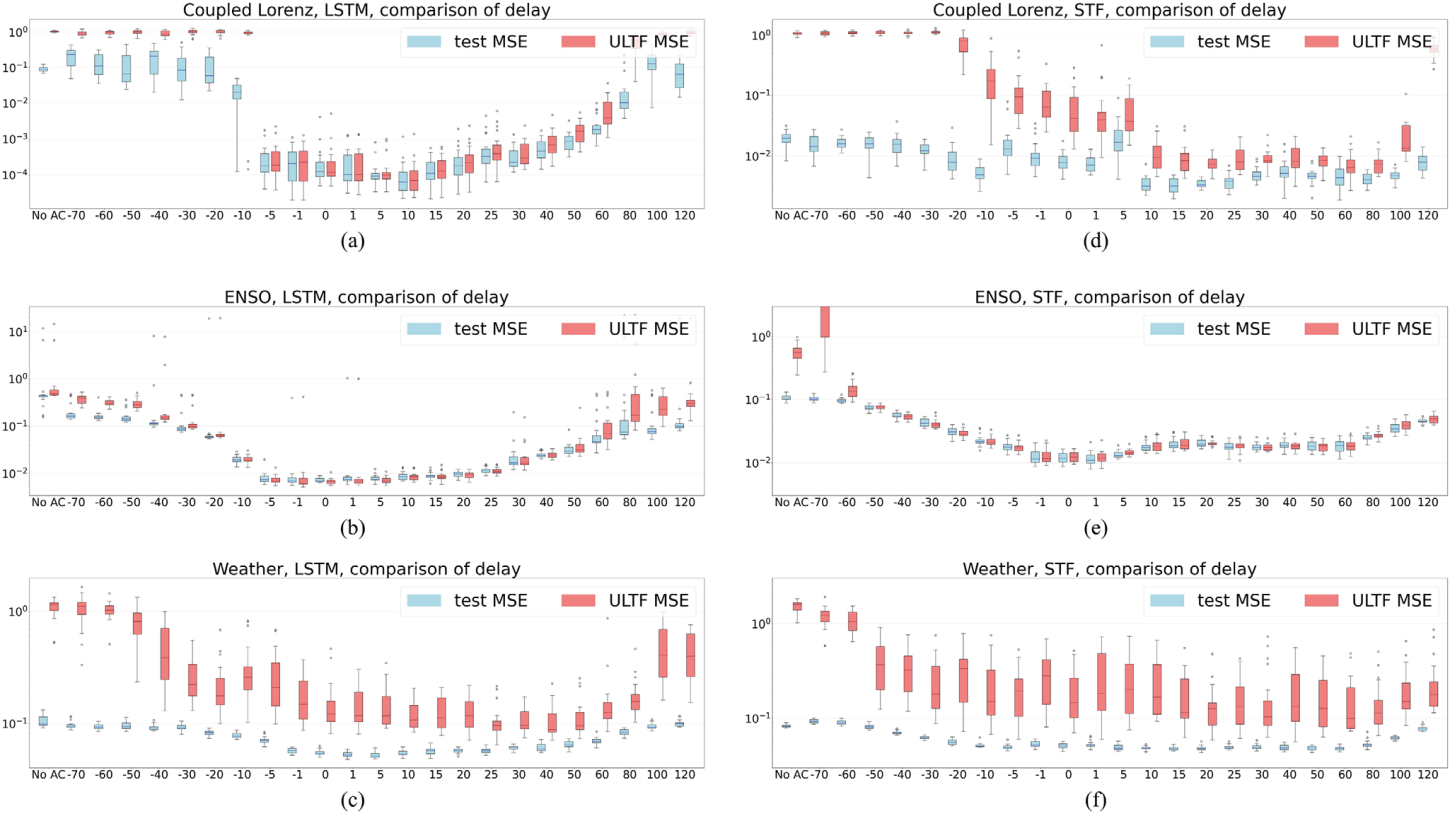
Sensitivity analysis of future info delay d. Compare the errors of performing multiple long‐term predictions on the test set and a single ultra‐long‐term forecasting using the entire test set. Results without any anticipated covariates serve as baselines. The context length s is set to 60. Each option is repeated 20 times.

In practice, observations of coupled systems often come from sensors distributed across different geographical locations, with varying modalities and even different sampling periods. Aligning the timesteps of time series is not a straightforward task. Potential misalignments between sequences require the choice of delay to be highly robust. The sensitivity of the paradigm to the future info delay d is systematically evaluated across various datasets and architectures, as illustrated by the error distributions in Figure 5. Instead of a sharp sensitivity to specific timing, the results exhibit a broad performance plateau – a wide range of d values where both standard testing MSE and ultra‐long‐term forecasting MSE remain consistently low and stable. A closer inspection reveals a functional distinction between the two backbones: while LSTM exhibits a preference for smaller delays to maintain accuracy, Spacetimeformer demonstrates superior resilience to larger delay variations, owing to its global attention mechanism that captures a comprehensive spatiotemporal field of view. Remarkably, the forecasting remains robust even when the delay approaches zero or becomes slightly negative, suggesting that the anticipated covariate paradigm does not rely on perfect temporal alignment. This characteristic significantly relaxes the stringent requirements for high‐precision timestamp synchronization across distributed sensors, making the paradigm highly applicable to real‐world systems with heterogeneous sampling periods.

#### 2.4.3 Comparison With State‐of‐the‐Art Models

To further demonstrate the effectiveness and simplicity of our anticipated covariate paradigm, we conduct a direct comparison with two recently proposed forecasting models that natively support exogenous inputs, XLinear [31] and TimeXer [32]. Both models have achieved state‐of‐the‐art performance on commonly used forecasting benchmarks, making them ideal candidates for evaluating whether our paradigm can be seamlessly integrated into diverse modern architectures. In their original formulations, XLinear and TimeXer accept exogenous variables only over the lookback window of length s. However, both models rely on embedding layers to process exogenous series. Specifically, TimeXer maps each exogenous series to variate tokens via a trainable linear projector, while XLinear projects both endogenous and exogenous features into a joint embedding space. These designs allow the input length of exogenous series to be extended easily, without altering the core architecture.

Under conditions similar to those described in Section Real‐world datasets, we conduct an additional analysis using zero matrices in place of the anticipated covariates comparing with unmodified ones to assess the contribution of such future information. For ultra‐long‐term forecasting, we adopt the same self‐iterative strategy as used for Spacetimeformer, using previous outputs as inputs for the next iteration with the same input and output shape. Considering it requires the predictor to output the complete system variables, we adopt the standard multivariate forecasting setting used in XLinear and TimeXer as our baseline. Letting X denote the full state variables in benchmark dataset, and E the subset used as anticipated covariates, “unmodified AC” mode can be described as ${\hat{X}}_{t+1:t+h}\;=\;N\left({\underset{︸}{X_{t-s:t}}}_{\text{endogenous channel}},\;{\underset{︸}{E_{t-s+d:t+h+d}}}_{\text{exogenous channel}}\right)$, where $E_{t-s+d:t+h+d}$ is known ground truth future state, “zero‐filled AC” mode can be described as ${\hat{X}}_{t+1:t+h}\;=\;N\left({\underset{︸}{X_{t-s:t}}}_{\text{endogenous channel}},\;{\underset{︸}{0}}_{\text{exogenous channel}}\right)$, and “standard M” mode can be described as ${\hat{X}}_{t+1:t+h}\;=\;N\left({\underset{︸}{X_{t-s:t}}}_{\text{endogenous channel}},\;{\underset{︸}{X_{t-s:t}}}_{\text{exogenous channel}}\right)$, where $X_{t-s:t}$ input to both channels is the prediction from the previous iteration.

**FIGURE 6:**
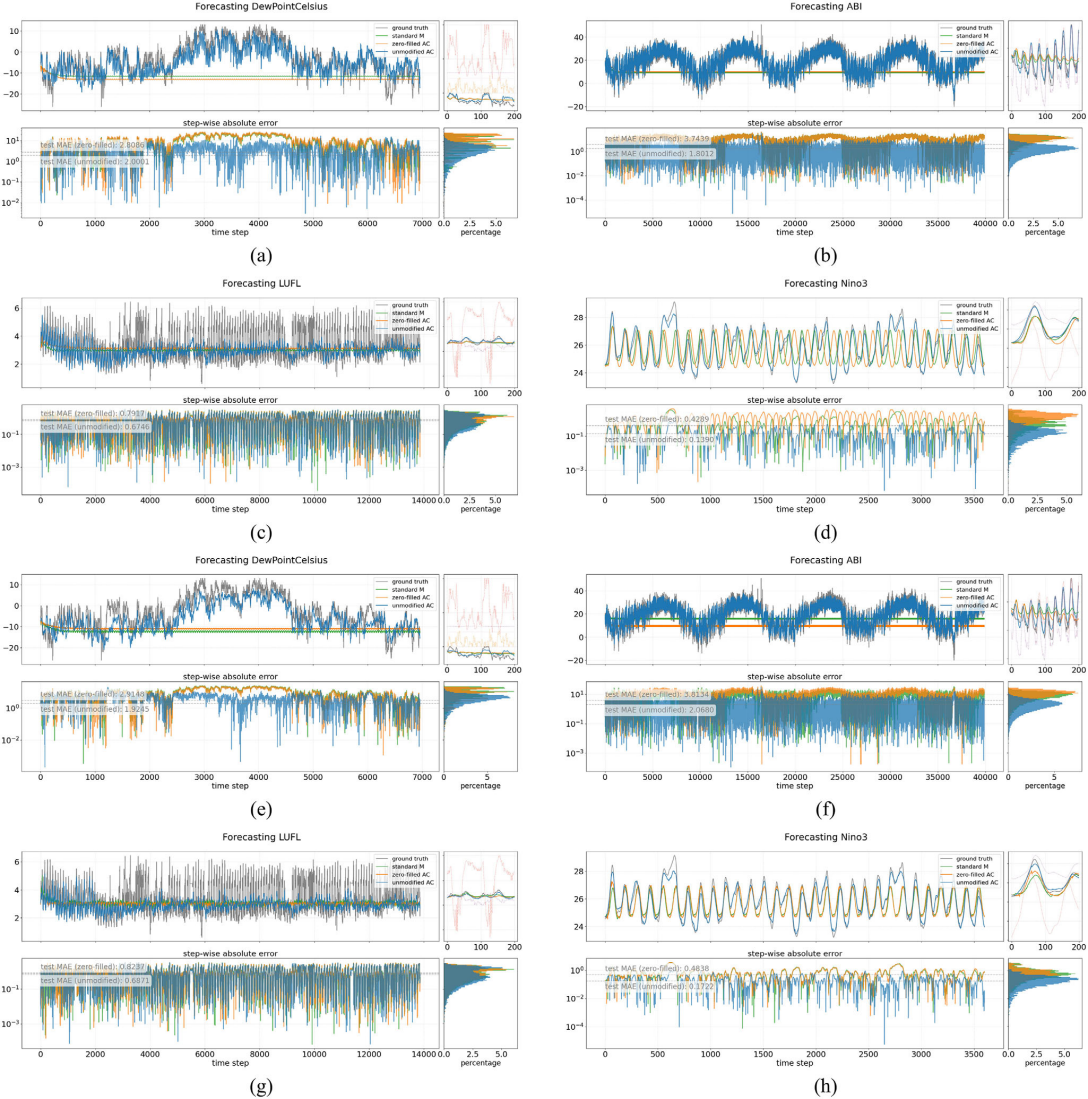
Performance comparisons with XLinear (a–d) and TimeXer (e–h). Anticipated covariates are replaced by array of zeros with the same shape while training, testing and ultra‐long‐term prediction to compare with unmodified ones. The first 200 time steps are detailed, with dash lined real anticipated covariates trajectories. The unnormalized test MAE values of target variables, plotted with horizontal dashed lines, serve as baselines for comparing step‐wise errors. All models are evaluated with a context length s∈[40,60], similar to the previous real‐world dataset experiments.

The ultra‐long‐term forecasting results are reported in Figure 6. When the model is provided with zero anticipated covariates, both XLinear and TimeXer quickly fall into periodic cycles and fail to track the ultra‐long‐term temporal evolution of the target variable. In contrast, with real anticipated covariates, the models accurately capture the overall trend and maintain low prediction errors over thousands of steps. The stark contrast between normal and zero‐filled anticipated covariates confirms that the improvement stems from the information content of the effect variable's future, rather than from a mere increase in input dimensionality. Moreover, this demonstrates that the anticipated covariates paradigm is not tied to a specific predictor but can be effectively combined with other recent architectures as long as they support exogenous inputs.

## 3 Conclusion

To summarize, this work revisits the task of time series forecasting with an inverse causal perspective of effect predicting cause and an inverse temporal perspective of future predicting present. We improve commonly used neural network forecasting algorithms by feeding future effect information into the forecasting model as covariates and validate it with extensive real‐world and synthetic data.

Accurate ultra‐long‐term forecasting remains highly challenging, and practical applications are rarely explored in existing literature. Despite the common perception that future information is difficult to obtain, we envision that the anticipated covariate paradigm will find broad applicability across numerous real‐world scenarios. Based on the theoretical characteristics of anticipated covariates, we identify several promising scenarios that are particularly well‐suited to this paradigm.

One natural setting for anticipated covariates is the presence of existing operational forecasting systems whose future outputs are already routinely available. For instance, numerical weather prediction models routinely produce high‐quality forecasts of wind fields, humidity, and pressure. These outputs may be directly repurposed to refine local temperature or precipitation predictions, achieving higher resolution or accuracy than the original forecasts alone. Similarly, hydrological models generate future water level or flow predictions for downstream stations, which might be used to infer upstream inflow patterns, thereby extending flood warning lead times beyond what conventional methods allow. In electricity markets, day‐ahead load and price forecasts are standard products. Incorporating them would help optimize generator dispatch and improve short‐term load forecasting accuracy.

Observability asymmetry presents another critical application domain, specifically when an effect is significantly more accessible or cost‐effective to measure than its upstream cause. By leveraging accessible signals as anticipated covariates, hidden states can be inferred without direct measurement. In medical diagnostics, wearable devices continuously record heart rate, skin conductance, and body temperature. These easily obtainable surface signals serve as effects of deeper physiological states such as blood glucose, hormone levels, or organ stress. In industrial maintenance, critical machinery such as turbines or engines often have internal components whose health is difficult to monitor directly. Yet surface‐mounted vibration sensors, acoustic sensors, and thermal cameras are inexpensive and easy to install. Their future readings may help predict remaining useful life of the core components. This asymmetry is particularly compelling when the cause itself is driven by unobservable factors. For instance, in environmental monitoring, industrial emissions or illegal discharges can be difficult to track in real time due to hidden operational changes, yet downstream water quality stations continuously record pollutant concentrations. Using future downstream concentrations would allow inference of upstream emission intensity, enabling targeted regulatory enforcement.

Furthermore, in situations where historical data are incomplete, continuously monitored related variables may help reconstruct the missing segments of a target time series. In paleoclimatology, tree‐ring sequences often contain gaps due to unfavorable growth conditions. Yet sediment layers, pollen records, and isotopic ratios from ice cores are often continuously preserved. These continuous proxies could offer a promising pathway for reliable reconstruction of the missing tree‐ring data, yielding a more complete picture of past climate variability. In infectious disease surveillance, early epidemic trajectories are frequently obscured by delayed reporting or under‐detection. However, syndromic surveillance data such as online search trends, over‐the‐counter medication sales, and emergency call volumes are recorded continuously and in near real‐time. Their future values might help infer the real infection curve, filling the early missing records and improving outbreak response.

Beyond ultra‐long‐term forecasting, the anticipated covariate paradigm may also offer practical benefits in applications where improving short‐ to medium‐term accuracy is the primary objective, as demonstrated by the improved test performance across all datasets in this study.

Ultimately, the significant guiding value of future effect information warrants a re‐evaluation of its role in predictive modeling, as it provides a decisive solution to the error accumulation typical of ultra‐long‐term horizons. While such information is traditionally perceived as difficult to acquire, our paradigm demonstrates that its groundbreaking performance justifies the pursuit of these covariates, especially in domains where effects are more observable or accessible than hidden causal drivers. By successfully leveraging these future‐past dependencies, this work not only pushes the boundaries of forecasting stability but also offers novel insights and research directions at the intersection of time series analysis and causal inference.

## 4 Methods

### 4.1 Preliminaries on Dynamical Systems

To provide a theoretical perspective and motivation for the anticipated covariates paradigm and address the underlying mechanisms of ultra‐long‐term forecasting, we briefly introduce key concepts from nonlinear dynamical systems and causality.

#### 4.1.1 Coupled Systems and Causality

Many real‐world systems consist of interacting components. In the study of complex systems, causality inference and time series forecasting are naturally connected. From a dynamical systems perspective, causality is frequently modeled through directional coupling. Consider a system with a cause variable $Y\in R^{n_{y}}$ and an effect variable $X\in R^{n_{x}}$. A unidirectional causal link Y→X implies that the time evolution of X is explicitly driven by the state of Y, while Y evolves independently of X. Formally, in discrete time, such a system can be written as

(2) $$\begin{array}{cc} Y_{t} & =f_{Y}\left(Y_{t-1}\right),\\ X_{t} & =f_{X}\left(Y_{t-1},X_{t-1}\right), \end{array}$$

where $f_{Y}:R^{n_{y}}\to R^{n_{y}}$ and $f_{X}:R^{n_{y}}\times R^{n_{x}}\to R^{n_{x}}$ are smooth maps. Because the effect variable X undergoes dynamic trajectory changes due to the influence of the cause variable Y, these changes inherently encode the state information of Y at past moments. This causal asymmetry is the key property exploited by our paradigm.

#### 4.1.2 Chaos and Lyapunov Exponents

A defining characteristic of many real‐world dynamical systems is chaos, representing an extreme sensitivity to initial conditions. This sensitivity is quantified by the Lyapunov exponents, which measure the average exponential rate of divergence or convergence of infinitesimally close trajectories in the state space. Consider a discrete‐time dynamical system $S_{t+1}=F\left(S_{t}\right)$ with state vector $S_{t}\in R^{n}$. For an infinitesimal perturbation $\delta S_{0}$ applied to the initial state, its evolution is governed by the linearized dynamics $\delta S_{t}=DF^{t}\left(S_{0}\right)\delta S_{0}$, where $DF^{t}$ denotes the derivative of the t‐fold composition of F. The Lyapunov exponent in the direction of $\delta S_{0}$ is defined as

$$\lambda \left(\delta S_{0}\right)=\lim_{t\to \infty }\frac{1}{t}\ln\frac{∥\delta S_{t}∥}{∥\delta S_{0}∥}.$$

For a generic initial state, the system possesses n Lyapunov exponents $\lambda_{1}\geq \lambda_{2}\geq \cdots \geq \lambda_{n}$ corresponding to the expansion/contraction rates along orthogonal directions in the tangent space. The maximal Lyapunov exponent $\lambda_{\max}=\lambda_{1}$ determines the overall predictability of the system. If $\lambda_{\max}>0$, the system is chaotic, meaning that nearby trajectories diverge exponentially over time. Consequently, for conventional autoregressive forecasting, which is ideally equivalent to performing open‐loop numerical integration of the system, the prediction error $∥e_{t+h}∥$ grows exponentially with the forecast horizon h as $∼e^{\lambda_{\max}h}$. This error accumulation inevitably leads to the failure of standard long‐term forecasting for chaotic systems.

#### 4.1.3 Taken' s Embedding Theorem and Its Generalizations

To reconstruct the hidden dynamics of a complex system from limited observations, we rely on the Takens' embedding theorem [33]. In its original formulation, Takens' theorem states that for a D‐dimensional compact manifold M and a smooth diffeomorphism F:M→M, if y:M→R is a smooth observation function, then for a generic pair (F,y), the delay‐coordinate map $\Phi :M\to R^{2D+1}$ defined by

$$\Phi \left(S\right)=\left(y\left(S\right),y\left(F^{1}\left(S\right)\right),y\left(F^{2}\left(S\right)\right),\cdots ,y\left(F^{2D}\left(S\right)\right)\right)$$

is an embedding. This means the reconstructed space preserves the topological properties of the original, unobserved attractor. In modern multivariate forecasting tasks, this principle is extended by the generalized Takens' embedding theorem [34]. Consider a system with a k‐dimensional observation function $\eta \left(S_{t}\right)=X_{t}\in R^{k}$. Under generic conditions, i.e., for almost all choices of the dynamics and observation function, if one constructs a delay vector using m future observations,

$$\Phi_{m}\left(S_{t}\right)=\left(X_{t+1},X_{t+2},\cdots ,X_{t+m}\right),$$

then this mapping is an embedding of the attractor A provided that the total number of scalar observations satisfies k·m>2dim(A). In our setting, $k=n_{x}$ and m=h+d, where h is forecasting length and d is future info delay.

#### 4.1.4 Observability in Dynamical Systems

In control theory and dynamical systems, observability refers to the ability to determine the full internal state of a system from knowledge of its external outputs over a finite time interval. Formally, for a system $S_{t+1}=F\left(S_{t}\right)$ with observation $\eta (S_{t})$, the system is said to be observable if the mapping from the initial state $S_{0}$ to the observation sequence $\left\{\eta \left(S_{0}\right),\eta \left(S_{1}\right),\cdots ,\eta \left(S_{T}\right)\right\}$ is injective, i.e., distinct initial states produce distinct observation sequences. The Takens' embedding theorem provides a powerful sufficient condition for observability in nonlinear systems: generically, a sufficiently long sequence of scalar observations suffices to embed the attractor. In the context of our coupled system Y→X, the effect variable X serves as the observation function $\eta \left(S_{t}\right)=X_{t}$.

### 4.2 Mechanism Analysis

Consider a noise‐free discrete‐time coupled dynamical system comprising a cause variable $Y_{t}\in R^{n_{y}}$ and an effect variable $X_{t}\in R^{n_{x}}$. The variables interact through unidirectional coupling Y→X. Conventional time series forecasting methods typically learn an autoregressive mapping $F:R^{s\times (n_{y}+n_{x})}\to R^{n_{y}+n_{x}}$, using a historical window $\left\{Y_{t-s:t},X_{t-s:t}\right\}$ to predict the next state $\left({\hat{Y}}_{t+1},{\hat{X}}_{t+1}\right)$ and iterating to produce multi‐step forecasts. This is ideally equivalent to performing open‐loop numerical integration of system Equation 2. Its error dynamics are governed by the system's maximum Lyapunov exponent $\lambda_{\max}$: the prediction error $∥e_{t+h}∥$ grows exponentially with the forecast horizon h as $∼e^{\lambda_{\max}h}$, leading to the doomed failure of long‐term prediction for chaotic systems with $\lambda_{\max}>0$.

The anticipated covariates paradigm proposed in this work reformulates the prediction problem. By incorporating the observed future sequence of the effect variable, $X_{t+1:t+h+d}$, as a known input, it constructs a conditional generative mapping $G:R^{s\times (n_{y}+n_{x})}\times R^{\left(h+d\right)\times n_{x}}\to R^{h\times n_{y}}$, such that

(3) $${\hat{Y}}_{t+1:t+h}=G\left(Y_{t-s:t},X_{t-s:t},X_{t+1:t+h+d}\right).$$

The feasibility and efficacy of this mapping are rooted in the observability theory of nonlinear dynamical systems. To elucidate the underlying mechanism, we express system Equation (2) in autonomous form. Define the state vector $S_{t}=\left(Y_{t},X_{t}\right)\in R^{n_{y}+n_{x}}$, with dynamics $S_{t}=F\left(S_{t-1}\right)$, where $F=(f_{Y},f_{X})$. The observed variable is $X_{t}$, corresponding to the observation function $\eta \left(S_{t}\right)=X_{t}$, i.e., a projection map. The relationship between the future observation sequence and the initial state is

(4) $$X_{t+k}=\eta \left(F^{\left(k\right)}\left(S_{t}\right)\right),\;k=1,2,\cdots ,h+d,$$

where $F^{\left(k\right)}$ denotes the k‐th iterate of F. Define the mapping $\Phi_{h+d}:R^{n_{y}+n_{x}}\to R^{\left(h+d\right)\times n_{x}}$ from the initial state $S_{t}$ to the future observation sequence

(5) $$\Phi_{h+d}\left(S_{t}\right)=\left(\eta \left(F\left(S_{t}\right)\right),\eta \left(F^{\left(2\right)}\left(S_{t}\right)\right),\cdots ,\eta \left(F^{\left(h+d\right)}\left(S_{t}\right)\right)\right).$$

According to the generalized Takens' embedding theorem and its extensions to multivariate observations, if F is a diffeomorphism, the dynamical system it generates is ergodic on a compact attractor $A\subset R^{n_{y}+n_{x}}$, and the observation function η is a generic smooth functional, then for almost all smooth η and a sufficiently large h+d>2dim(A), the mapping $\Phi_{h+d}$ is an embedding on A, i.e., injective and with an injective differential everywhere. This implies that the initial state $S_{t}$ can be uniquely and smoothly reconstructed from the observation sequence $X_{t+1:t+h+d}$, further enhancing the prediction of $Y_{t+1:t+h}$ by learning this inverse mapping through neural network G.

From the perspective of system observability, the key requirement is that the future observation sequence can uniquely determine the trajectory of the cause variable Y. This does not necessitate the observation function η itself to be invertible but requires that the composite mapping $\Phi_{h+d}$ be distinguishable in the Y component. Specifically, defining the projection $\pi_{Y}\left(S_{t}\right)=Y_{t}$, we need $\pi_{Y}\circ \Phi_{h+d}^{-1}$ to exist and be Lipschitz continuous. This is equivalent to the observability condition in the Y direction: there exist constants L>0 and integer m>0 such that for any two states $S_{t},S_{t}^{\prime }\in A$, if $\Phi_{m}\left(S_{t}\right)=\Phi_{m}\left(S_{t}^{\prime }\right)$, then $∥Y_{t}-Y_{t}^{\prime }∥\leq L∥\Phi_{m}\left(S_{t}\right)-\Phi_{m}\left(S_{t}^{\prime }\right)∥$. Under the assumption of causality Y→X, this condition is typically satisfied because differences in Y are amplified through $f_{X}$ and manifested in the trajectory of X.

From an optimization viewpoint, conventional methods minimize open‐loop integration error, whereas the anticipated covariate paradigm minimizes trajectory fitting error under the constraint of future observations. This is equivalent to solving a boundary value problem: given a historical trajectory segment and a future observation segment, find a system trajectory that simultaneously satisfies both the dynamical constraints and the observational constraints. The future observation sequence provides $h\cdot n_{x}$ scalar constraints, which implicitly determine the trajectory of the cause variable through the system dynamics, thereby substantially reducing the solution space.

For scenarios involving unobserved driving factors (e.g., Z→Y→X), as long as the coupling Y→X is sufficiently strong, the future X sequence can still provide adequate information about Y. This is because the future evolution of X is primarily driven by Y, with the influence of Z indirectly reflected through Y. In this case, the mapping $\Phi_{h+d}$ remains an embedding on the subspace (Y,X), and the neural network can learn the mapping from the X sequence to the Y sequence without explicitly modeling Z.

In summary, the effectiveness of the anticipated covariate paradigm does not rely on the local invertibility of the observation equation but depends on the overall observability of the coupled system in the temporal dimension–a property jointly determined by the dynamics $f_{Y},f_{X}$, the observation function η, and the delay length d. This method transforms the long‐term prediction problem from an initial‐value‐sensitive, open‐loop integration task into a state reconstruction problem utilizing future observations as strong constraints, thereby fundamentally suppressing the exponential accumulation of errors. It provides a theoretically sound and practically viable new pathway for ultra‐long‐term time series forecasting.

### 4.3 Dataset Processing

For each time series dataset $X\in R^{T\times N}$ with given parameters: future information delay d, anticipated covariates dimension set E⊂{1,2,⋯,N}, input sequence length s, and output sequence length h, the data preprocessing proceeds as follows: Extract the anticipated covariate sequence $E\in R^{\left(T-d\right)\times \left|E\right|}$ by retaining temporal components from the d‐th timestep onward and spatial dimensions pecified in E, i.e., E=X[d:,E]. Trim the last d timesteps of X to ensure temporal alignment between X and E with a timestep length of T−d. Allocate the first 70%+10% of aligned timesteps for training/validation and the remaining 20% for testing. Apply a synchronized sliding window of width s+h to X and E, generating slices $x\in R^{(s+h)\times N}$ (from X) and $e\in R^{\left(s+h\right)\times \left|E\right|}$ (from E). First s timesteps of x serve as the input sequence while subsequent h timesteps serve as the expected output. The complete sequence e provides anticipated information for this sample slice. For ultra‐long‐term forecasting tasks, the last 20% timesteps of X and E are treated as a unified window, where the initial s steps of x serve as input and all remaining steps constitute the prediction target, that is, s remains constant and h=(T−d)×0.2−s. While maintaining comparisons of overall prediction errors, we concurrently compute metrics focused exclusively on target variables, given that partial future states have been incorporated as model inputs.

Anticipated covariates are distinguished from conventional ones in that they represent future states of specific dimensions within the predicted system. Despite this conceptual difference, they are formally treated as standard covariates during implementation.

### 4.4 Neural Network Predictors With Anticipated Covariates

For the basic architecture of self‐iterative sequence forecasting with an “input ‐ neural network black box M ‐ output” framework, where the output is recursively used as the next input, differentiating the dimensions of inputs and outputs enables convenient utilization of covariate information. This is achieved by concatenating covariates with the previous output to form the complete input. That is: ${\hat{x}}_{t}=M\left(\mathrm{Concat}\left[e_{t-1},{\hat{x}}_{t-1}\right]\right),t=1,2,\text{…},h$. As shown in the RNN‐based predictor part of Figure 1, we demonstrate this approach by augmenting the conventional LSTM structure.

The LSTM network is a specialized type of RNN designed to model long‐term dependencies in sequential data [35]. An LSTM core employs three gating mechanisms – input gate ($i_{t}$), forget gate ($f_{t}$), and output gate ($o_{t}$) – along with a cell state ($c_{t}$) and a hidden state ($h_{t}$) to regulate information flow. For each timestep t, the input $x_{t}$ and the previous hidden state $h_{t-1}$ are processed through these gates as:

(6) $$\begin{array}{cc} i_{t} & =\sigma (W_{ii}x_{t}+b_{ii}+W_{hi}h_{t-1}+b_{hi}),\\ f_{t} & =\sigma (W_{if}x_{t}+b_{if}+W_{hf}h_{t-1}+b_{hf}),\\ o_{t} & =\sigma (W_{io}x_{t}+b_{io}+W_{ho}h_{t-1}+b_{ho}),\\ g_{t} & =\tanh(W_{ig}x_{t}+b_{ig}+W_{hg}h_{t-1}+b_{hg}),\\ c_{t} & =f_{t}⊙c_{t-1}+i_{t}⊙g_{t},\\ h_{t} & =o_{t}⊙\tanh\left(c_{t}\right), \end{array}$$

where the W terms denote weight matrices, the b terms denote bias vectors, σ is the sigmoid function, and ⊙ is the element‐wise product. The input gate determines how much new information to incorporate. The forget gate controls how much of the previous cell state to retain. The output gate then computes the hidden state, which serves as the output for the current timestep. The cell candidate $g_{t}$ represents the potential new information to be added to the cell state and scaled by the input gate to determine how much of $g_{t}$ is actually incorporated into cell state. In a multilayer LSTM, the input $x_{t}^{\left(l\right)}$ of the l‐th layer (l≥2) is the hidden state $h_{t}^{\left(l-1\right)}$ of the previous layer. The forecasting output of a single iteration is obtained after applying an affine linear transformation to the hidden state of the last layer: ${\hat{x}}_{t+1}=h_{t}^{L}A^{⊤}+b$.

Teacher forcing technique consists of using the ground truth data as input for each timestep and is proved to be efficient in natural language processing related tasks. However, during inference, when predicting multiple timesteps ahead, the model has to substitute unknown preceding values with its own predictions. Teacher forcing therefore creates a mismatch between training and inference conditions. For consistency between training and prediction, we implement a training procedure without teacher forcing [36]. For any sequence‐to‐sequence forecasting task, within the input sequence, the known system state is used as input; within the prediction sequence, the last predicted state is used as input.

Simply replace the input of such conventional LSTM with vectors concatenated by state variables $x_{t}$ and covariates $e_{t}$, we denote one single LSTM iteration with anticipated covariates as

(7) $$\begin{array}{cc} h_{t},c_{t} & =\mathrm{LSTM}(x_{t},e_{t},h_{t-1},c_{t-1}),\\ {\hat{x}}_{t+1} & =\mathrm{Linear}(h_{t}). \end{array}$$

For an input sequence ${\left\{x_{t}\right\}}_{t=-s}^{0}$, an anticipated covariates sequence ${\left\{e_{t}\right\}}_{t=-s}^{h}$ and an expected sequence ${\left\{y_{t}\right\}}_{t=1}^{h}$ within a window slice, the forecast output ${\left\{{\hat{x}}_{t}\right\}}_{t=1}^{h}$ is obtained by

(8) $$h_{t},c_{t}=\mathrm{LSTM}\left(x_{t},e_{t},h_{t-1},c_{t-1}\right),$$

for t=−s,−s+1,…,0, and

(9) $$\begin{array}{cc} {\hat{x}}_{t+1} & =\mathrm{Linear}(h_{t})\\ h_{t+1},c_{t+1} & =\mathrm{LSTM}({\hat{x}}_{t+1},e_{t+1},h_{t},c_{t}), \end{array}$$

for t=0,1,…,h−1. The hidden state h and cell state c are both initialized as zero matrices. MSE between ${\left\{y_{t}\right\}}_{t=1}^{h}$ and ${\left\{{\hat{x}}_{t}\right\}}_{t=1}^{h}$ serves as loss and Adam algorithm is used to update the learnable variables.

The Transformer architecture [37], introduced for sequence‐to‐sequence tasks, employs attention mechanisms to dynamically model dependencies between input tokens. Each token is represented by a query, key, and value vector. The attention scores, computed via scaled dot‐product similarity between queries and keys, form an adjacency matrix that governs information flow between tokens. This matrix is normalized using softmax and used to compute weighted sums of value vectors, enabling the model to capture long‐range interactions. In encoder‐decoder frameworks, the encoder processes the context sequence through self attention, while the decoder generates predictions by attending to both its own outputs (self attention) and the encoder's representations (cross attention). Transformers' permutation invariance is addressed through learnable positional embeddings, which encode token order. For an input sequence $x\in R^{l_{x}\times d_{x}}$, self attention enables it to dynamically focus on its own elements to capture long‐range dependencies through Query (Q), Key (K), and Value (V) by

(10) $$\begin{array}{c} Q=xW^{Q},K=xW^{K},V=xW^{V},\\ A\left(x,x\right)=\mathrm{softmax}\left(\frac{QK^{⊤}}{\sqrt{d_{x}}}\right),\\ \mathrm{Attention}(x,x)=A(x,x)V, \end{array}$$

where the scaling factor $\sqrt{d_{x}}$ prevents gradient instability caused by large dot‐product magnitudes. Cross‐attention allows the decoder to attend to the encoder's input sequence x while generating the output sequence y. Given contextual representation of x ‐ encoder output $h_{\mathrm{enc}}$ and partially generated output sequence ‐ decoder input $y_{\mathrm{dec}}$, the decoder generates Q,K,V just like self attention.

(11) $$\begin{array}{c} Q=y_{\mathrm{dec}}W^{Q},K=h_{\mathrm{enc}}W^{K},V=h_{\mathrm{enc}}W^{V},\\ A_{\mathrm{cross}}\left(y_{\mathrm{dec}},h_{\mathrm{enc}}\right)=\mathrm{softmax}\left(\frac{QK^{⊤}}{\sqrt{d_{x}}}\right),\\ \mathrm{CrossAttention}\left(y_{\mathrm{dec}},h_{\mathrm{enc}}\right)=A_{\mathrm{cross}}\left(y_{\mathrm{dec}},h_{\mathrm{enc}}\right)V. \end{array}$$

Spacetimeformer [24] reformulates multivariate time series forecasting by transforming inputs into an elongated spatiotemporal sequence. For a window slice with N variables and s+h timesteps, the input is reshaped from (s,N) to (s×N,1), where each token isolates a single variable at a specific timestep. This flattened sequence allows the Transformer to jointly model interactions across space (variables) and time (timesteps) through a unified attention mechanism. Unlike traditional methods that group variables per timestep into a single token, Spacetimeformer treats each variable‐timestep pair as an independent token, enabling dynamic and context‐dependent spatial relationships to emerge purely from data, without relying on predefined graphs.

By constructing separate instances for the encoder and decoder, the transformer adapts to two sequences of different lengths, that is, two sequences of different dimensions since the dimensions have been flattened. This framework naturally accommodates differentiated input‐output dimensions, enabling the concatenation of forecasting variables and anticipated variables for prediction in a manner similar to LSTM. To demonstrate more ways of leveraging expected information, we adopt an approach where covariates are independently embedded. In contrast, this independent method of mapping anticipated covariates into high‐dimensional vectors provides richer mapping strategy options, without being constrained to align with the system state representation, which helps tremendously in dealing with anticipated covariates taking a different form than the forecasting variables.

The Time2Vec method [38] exhibits outstanding performance in extracting temporal features, particularly seasonal patterns (e.g., daily, weekly, or annual cycles). By adaptively learning frequency and phase parameters through sine‐based transformations while retaining linear components for temporal progression, Time2Vec eliminates the reliance on manual feature engineering and achieves robust generalization. We find its inherent capability to jointly model periodic behaviors and non‐periodic trends ensures broad applicability across the diverse real‐world datasets explored in this work. Given that numerous real‐world datasets exhibit periodic characteristics, we employ the Time2Vec layers–inspired by its application in Spacetimeformer for encoding time information–to extract features from anticipated covariates. Benefiting from Time2Vec's dual capability, we demonstrate its applicability to the diverse real‐world data discussed in this study, as validated by experimental results presented in earlier sections.

The overall structure of Spacetimeformer with anticipated covariates is shown in the transformer‐based predictor part of Figure 1. Flattening the dimensions transforms a sequence‐to‐sequence problem of length s+h into an (s+H)×N one, where an increase in N results in greater learning complexity. Adding a local attention layer where each token focuses on the time step of its own spatial variable before the global attention layer helps to alleviate this challenge. The tokens would attend to each token in the sequence of their own variables and then to each token in the entire spatio‐temporal global sequence. Refer to Spacetimeformer [24] for more details on deployments such as high performance attention.

## Author Contributions

S.Y.L. conceived the idea; J.T.Z. and S.Y.L. designed the research; J.T.Z. performed the research; J.T.Z., Y.F.L., R.X.H., Z.X.G., and S.Y.L. analyzed the data and wrote the paper.

## Conflicts of Interest

The authors declare no conflicts of interest.

## Data Availability

The data that support the findings of this study are available from the corresponding author upon reasonable request.
